# Genome-wide identification, expression and functional analysis of glutathione *s*-transferase family members in *Quercus dentata* under heavy metal stresses

**DOI:** 10.3389/fpls.2025.1641553

**Published:** 2025-09-09

**Authors:** Jingjing Sha, Xiangyue Wu, Ao Shen, Xin Pang, Wenbo Wang, Pingsheng Leng, Zenghui Hu, Yazhou Zhao, Xiangfeng He

**Affiliations:** College of Landscape Architecture, Beijing University of Agriculture, Beijing, China

**Keywords:** glutathione S-transferases, *Quercus dentata*, heavy metal, cadmium, lead, gene family

## Abstract

Glutathione *S*-transferases (GSTs), a superfamily of multifunctional enzymes, are involved in plant growth, development, and response to biotic and abiotic stresses. In this study, 86 members of the GST family, denoted *QdGST*, were identified in the *Quercus dentata* genome and found to be distributed among six of the GST classes, with the majority in the tau class, followed by the lambda and phi classes. This uneven distribution of *QdGST* genes was observed across 11 chromosomes. Thirty-one tandem and seven segmental duplication events were found to have contributed to the expansion of the QdGST family. Moreover, a total of 29 categories of *cis*-acting elements were identified in the promoters of the *QdGST* genes, most of which were involved in defense and stress responses. RNA sequencing analysis revealed that most *QdGST* genes displayed tissue-specific expression patterns, and that cadmium or lead treatment induced the expression of 31 of them, most of which belonged to the tau class. Quantitative real-time PCR analysis confirmed the expression of cadmium- and lead-induced *QdGST* genes, with *QdGSTU20* and *QdGSTU36* in particular showing strong upregulation. *QdGSTU36* also enhanced yeast growth under cadmium and lead stresses when expressed in yeast. These findings lay a crucial foundation for further work to clarify the biological functions of *QdGST* genes associated with heavy metal tolerance in *Q. dentata*.

## 1 Introduction

The glutathione *S*-transferase (GST) superfamily comprises a group of multifunctional enzymes (EC 2.5.1.18) that are widely distributed across diverse living organisms, ranging from bacteria and fungi to plants and animals (Wang et al., 2023). The amino acid sequences of GSTs differ greatly among different subfamilies, but their overall structures remain remarkably similar (Duan et al., 2022). Typically, a GST protein contains two distinct functional regions: an N-terminal glutathione-binding site (G-site) and a C-terminal substrate binding site (H-site) (Edwards and Dixon, 2005). The G-site is highly conserved, whereas the H-site shows considerable variation, enabling binding of a variety of different substrates (Zhao et al., 2021). These two domains are in close proximity to each other in the three-dimensional structure and form catalytic sites with specific functions in different subcellular locations (Zhao et al., 2021). In general, GSTs facilitate the conjugation of reduced glutathione to diverse hydrophobic and electrophilic substrates (Mo et al., 2023).

GSTs have vital role in plant growth and development (Gong et al., 2005), as well as transport and metabolism of secondary compounds (Dixon et al., 2010) and response to various stresses including exposure to bacterial and fungal pathogens (Frova, 2006; Perperopoulou et al., 2018), cold (Kayum et al., 2018; Song et al., 2021), chemical toxicity (Xu et al., 2016), salinity (Xu et al., 2016), drought (Chen et al., 2012; Xu et al., 2016), ultraviolet radiation (Liu and Li, 2002), and heavy metals (Gao et al., 2020; Jing et al., 2020). Plant GSTs can be divided into 14 classes on the basis of protein sequence, gene structure, gene function, and immunological characteristics (Wang et al., 2020; Zhao et al., 2021; Liu et al., 2023). The phi (GSTF), tau (GSTU), lambda (GSTL), and dehydroascorbate reductase (DHAR) classes are exclusive to plants, with phi and tau being the most prevalent (Wang et al., 2020).

Developments in sequencing technology and the associated reductions in the costs of sequencing have led to the discovery of increasing numbers of GSTs in both model and non-model plants, including *Arabidopsis* (Sappl et al., 2009), rice (Jain et al., 2010), poplar (Lan et al., 2009), tomato (Islam et al., 2017), sweet potato (Ding et al., 2017), pumpkin (Kayum et al., 2018), *Brassica rapa* (Khan et al., 2018), *Physcomitrella patens* (Liu et al., 2013), *Gossypium hirsutum* (Xu et al., 2017), *Cucumis melo* (Wang et al., 2020), *Malus* (Fang et al., 2020), *Capsicum annuum* (Islam et al., 2019), maize, and soybean (McGonigle et al., 2000). However, no such research has yet focused on GSTs in plants of the Fagaceae family, despite their ecological and economic significance of these species.

GSTs participate in resistance to heavy metal stresses in various organisms, including plants (Kumar et al., 2013; Liu et al., 2013; Lim et al., 2005; Ezaki et al., 2000), animals (Xu et al., 2016; Park et al., 2019; Takenaka et al., 2014; Nair and Choi, 2011), and fungi (Shen et al., 2015; Dixit et al., 2011). For instance, the fungal *TvGST* in *Trichoderma virens* has a role in tolerance to cadmium (Cd) stress (Dixit et al., 2011), whereas tobacco GST gene *parB* confers resistance to copper (Cu) and aluminum (Al) in *Arabidopsis* (Ezaki et al., 2000). Overexpression of the *Nt107* gene from the tobacco tau subfamily has been reported to result in accumulation of Cu in *Dianthus superbus* (Lim et al., 2005); similarly, overexpression of *PpGST*, a zeta GST gene from *Pyrus pyrifolia*, enhanced the tolerance of transgenic tobacco lines to Cd stresses (Liu et al., 2013), and *Arabidopsis* with heterologous expression of rice lambda-class *OsGSTL2* exhibited tolerance to arsenic (As), Cd, and chromium (Cr) treatments (Kumar et al., 2013). Overexpression of *OsGSTU6* in rice reduces accumulation of Cd in leaves and enhances the tolerance of the plant to Cd stress; conversely, reduced *OsGSTU6* expression levels are associated with Cd accumulation and diminished tolerance to heavy metal stress (Jing et al., 2020). The expression of tau- and theta-class *GSTs* can be induced by Cd, Cr, and lead (Pb) stresses in radish (Gao et al., 2020); some tau GST genes can be induced by Cd and As stresses in rice (Moons, 2003; Ahsan et al., 2008; Norton et al., 2008; Lin et al., 2013); and Cd treatment can trigger GST gene expression in wheat and maize (Mauch and Dudler, 1993; Marrs and Walbot, 1997). Moreover, accumulation of GST proteins has been observed in poplar and soybean under Cd stress (Sobkowiak and Deckert, 2006; Kieffer et al., 2009).

*Quercus* is the largest genus in the Fagaceae family and contains the most abundant and economically important woody plants (Wang et al., 2023). *Quercus* species can tolerate multiple heavy metal stresses, such as cobalt (Co), Pb, Cu, zinc (Zn), Cd, antimony (Sb), and nickel (Ni), and thus contribute significantly to the restoration of environments affected by heavy metal pollution (Gogorcena et al., 2011; Shi et al., 2017, Shi et al., 2019). However, the molecular mechanism underlying this heavy metal tolerance in *Quercus* plants remains unclear, and no research on GST genes in this genus has previously been published. *Quercus dentata* is a key species in northern China. Here, we systematically evaluated the potential roles of *Quercus* GSTs in heavy metal tolerance using high-quality whole-genome and transcriptome data for *Q. dentata* (Wang et al., 2023). We identified 86 *QdGST* genes and characterized their protein products using bioinformatics techniques; then, we analyzed their expression patterns under exposure to different heavy metals (Cd and Pb) using quantitative real-time PCR (qRT-PCR). Candidate *QdGST* genes with potential roles in heavy metal tolerance were expressed in yeast to confirm their effects. The results demonstrated that several *QdGST* genes of the tau subfamily contributed to the heavy metal tolerance of *Q. dentata*. These findings provide new insight into the evolutionary history and functional roles of *QdGST* genes and will provide a valuable reference for breeding efforts to develop plants with heavy metal resistance.

## 2 Materials and methods

### 2.1 Plant materials, growth condition and treatment

A mixture of *Q. dentata* seeds and sand with a humidity of 60% was stored in a refrigerator at 4°C for 3 weeks and then soaked in water at 40°C three times at room temperature (Zhang et al., 2023). After treatment, the *Q. dentata* seeds were sown in a 1:1 vermiculite-peat substrate and cultivated at 25°C/20°C with a 16 h/8 h light/dark cycle at 60% humidity. The 1/2 Hoagland nutrient solution was prepared without calcium nitrate according to the manufacturer’s protocol (Hope Bio-Technology Corporation Ltd., Qingdao, China). Seedlings of the same size and state were transplanted into 1/2 Hoagland nutrient solution and grown under the same conditions as used for sowing. Cd-grown and Pb-grown seedlings were treated with 200 mg/L CdSO_4_·8/3H_2_O and 1000 mg/L PbCl_2_, respectively (Gao et al., 2020). Seedlings cultivated in standard 1/2 Hoagland solution were used as controls. Roots, stems, and leaves of Cd-grown, Pb-grown and control seedlings were separately harvested for RNA extraction at 0 h and 24 h after transplantation. For each treatment, three culture bottles were used, with one seedling per bottle.

### 2.2 Characterization and identification of GST genes in *Q. dentata*

Genomic data of *Q. dentata* were obtained from the National Genomics Data Center (https://ngdc.cncb.ac.cn/) under BioProject PRJCA013491 with accession number GWHBRAD00000000) (Wang et al., 2023). To locate *QdGST* genes within the genome, a BLAST search was conducted with GST genes from *Arabidopsis thaliana* as the query sequences. In addition, *Arabidopsis* GST protein sequences were retrieved from the TAIR database (https://www.arabidopsis.org/) and used to screen for *Q. dentata* GSTs on the basis of sequence similarity (BLASTP; e-value ≤ 10^–10^). Hidden Markov model profiles for the GST_N (PF02798) and GST_C (PF00043) domains were obtained from the Pfam database and used to identify GST domains. *Q. dentata* protein sequences identified in homology searches were subsequently analyzed using the NCBI conserved domain database, HMMER, Pfam, and SMART (Letunic et al., 2004). The resulting QdGSTs were classified on the basis of their homology to *Arabidopsis* GSTs using a previously described standard method (Ghangal et al., 2020).

### 2.3 Phylogenetic analysis

Sequence alignment of GST proteins from *Arabidopsis* and *Q. dentata* was performed using the MUSCLE wrapper in TBtools with default parameters to examine evolutionary relationships among the proteins (Chen et al., 2020). A maximum likelihood phylogenetic tree was built using the ‘One Step Build a ML Tree’ feature in TBtools, applying the JTT substitution model with a 95% site coverage cut-off. Node confidence was evaluated using 1000 bootstrap replicates. GST classes were visualized using with distinct colors for clarity.

### 2.4 Amino acid characteristic analysis of QdGSTs

The characteristics of QdGST proteins, including molecular weight, isoelectric point, and grand average of hydropathicity, were analyzed using the ExPasy tool (http://web.expasy.org/). Their subcellular localizations were predicted with Cell-PLoc 2.0 (http://www.csbio.sjtu.edu.cn/bioinf/Cell-PLoc-2/).

### 2.5 Genome structure, chromosomal localization, gene duplication, and collinearity analysis

The chromosomal locations of 86 *QdGST* genes were obtained from a genome annotation file and mapped to chromosomes using TBtools (Chen et al., 2020). Genomic data for *A. thaliana*, *C. annuum*, *Vitis vinifera*, *Triticum aestivum*, and *Oryza sativa* were obtained from EnsemblPlants (http://plants.ensembl.org); those for *Quercus mongolica* were obtained from the NCBI Sequence Read Archive under accession codes PRJNA609556 and PRJNA607679. All the genomic data were analyzed for collinear relationships using the ‘One Step MCScanX’ feature in TBtools with default settings (Chen et al., 2020).

### 2.6 Analysis of *QdGST* gene structure, conserved motifs, and domains

Structural information regarding the *QdGST* genes was obtained from a GFF file and used to visualize the conserved domains and exon–intron organization with TBtools. Conserved motif sequences and types of *QdGST* genes were analyzed using MEME Suite 5.5.3 (http://meme-suite.org/tools/meme) with the following parameters: motif site distribution = any number of repetitions; and maximum motif number = 10. The structures and motif distributions of *QdGST* genes were grouped according to a phylogenetic tree and visualized using TBtools (Chen et al., 2020).

### 2.7 Analysis of *cis*-acting elements of *QdGST* gene promoters

Promoter regions (2,000 bp upstream of the translation initiation site) were extracted from all *QdGST* genomic sequences using TBtools (Chen et al., 2020). Potential *cis*-regulatory elements within these regions were identified by searching the PlantCARE database (http://bioinformatics.psb.ugent.be/webtools/plantcare/html/) with default settings.

### 2.8 Expression analysis of *QdGST* genes using RNA sequencing

To investigate the tissue-specific expression of the *QdGST* genes, we obtained RNA expression data for *Q. dentata* from the National Genomics Data Center (accession code GWHBRAD00000000). For analysis of the expression of *QdGST* genes under Pb and Cd stresses, samples were collected using the method described above. Total RNA extraction and mRNA library construction and sequencing were performed according to the methods described by Chen et al. (2022). All RNA-seq data have been submitted to the Genome Sequence Archive (accession CRA013085) of the National Genomics Data Center (CNCB-NGDC Members and Partners, 2022). Expression levels were normalized to FPKM (fragments per kilobase million), and a heatmap was generated by applying the logarithmic transformation log_10_(FPKM+1) using TBtools.

### 2.9 qRT-PCR analysis

Total RNA was extracted from the samples using a SteadyPure Plant RNA Extraction Kit (Accurate Biotechnology, Hunan, China) according to the protocol from manufacturer. Complementary DNA (cDNA) was synthesized using *Evo M-MLV* RT Premix for qPCR (Accurate Biotechnology). The primers for the qRT-PCR experiments were designed using Primer3plus (https://www.primer3plus.com/) and are listed in **Supplementary Table S3**. The *CaCs* gene was selected as the internal reference gene for roots, whereas the *EF1-α* gene was used for stems and leaves (**Supplementary Table S3**). Subsequently, the qPCRs were performed on a Bio-Rad CFX96 Touch Real-Time PCR Detection System (USA) using an SYBR Green Premix ProTaq HS qPCR Kit (Accurate Biotechnology) under the following conditions: 95°C for 30 s, followed by 40 cycles of 95°C for 15 s and 60°C for 30 s. The 2^−ΔΔCt^ method with three technical replicates per sample was used to calculate relative gene expression (Livak and Schmitten, 2001).

### 2.10 Heterologous expression of *QdGST* in yeast

Gene-specific primers (**Supplementary Table S3**) for *QdGSTU20* and *QdGSTU36* were used to amplify their coding DNA sequence regions. To enable expression in yeast (*Saccharomyces cerevisiae*), cDNAs for *QdGSTU20* and *QdGSTU36*, obtained through PCR amplification, were cloned into the KpnI-XbaI restriction sites of the pYES2.0 plasmid. A transformation kit (Huayueyang Biotechnology, China) was used to introduce the empty plasmid and recombinant vector into *S. cerevisiae* INVSc1. Jiang’s method with minor modifications was used to evaluate the metal resistance of the transgenic yeast (Jiang et al., 2024). Yeast harboring either the recombinant plasmid or the empty plasmid was cultured in liquid medium (SC-U/Glu) and incubated at 28°C and 200 rpm until the optical density at 600 nm reached 1.0. Subsequently, serial ten-fold dilutions were performed using sterile water, and 2-μL aliquots from each dilution were dropped onto solid SC-U/Gal induction medium supplemented with various heavy metals (10 μM CdSO_4_·8/3H_2_O, 3 mM PbCl_2_, or 15 mM MnSO_4_·H_2_O) or control medium (no additional metal ions). Three independent biological replicates were included in each treatment. Growth phenotypes were recorded after 3 days of incubation at 28°C.

### 2.11 Statistical analysis

GraphPad Prism 9.4.1 was selected for statistical analysis and graph generation. In figures showing the results of qPCR analyses, error bars represent the standard deviation from three independent biological replicates. Statistical analysis was performed using one-way analysis of variance and *post hoc* least significant difference tests to identify differences among group means, at a significance level of 0.05.

## 3 Results

### 3.1 Identification and phylogenetic and characteristic analysis of the *GSTs* in *Q. dentata*

**Figure 1:**
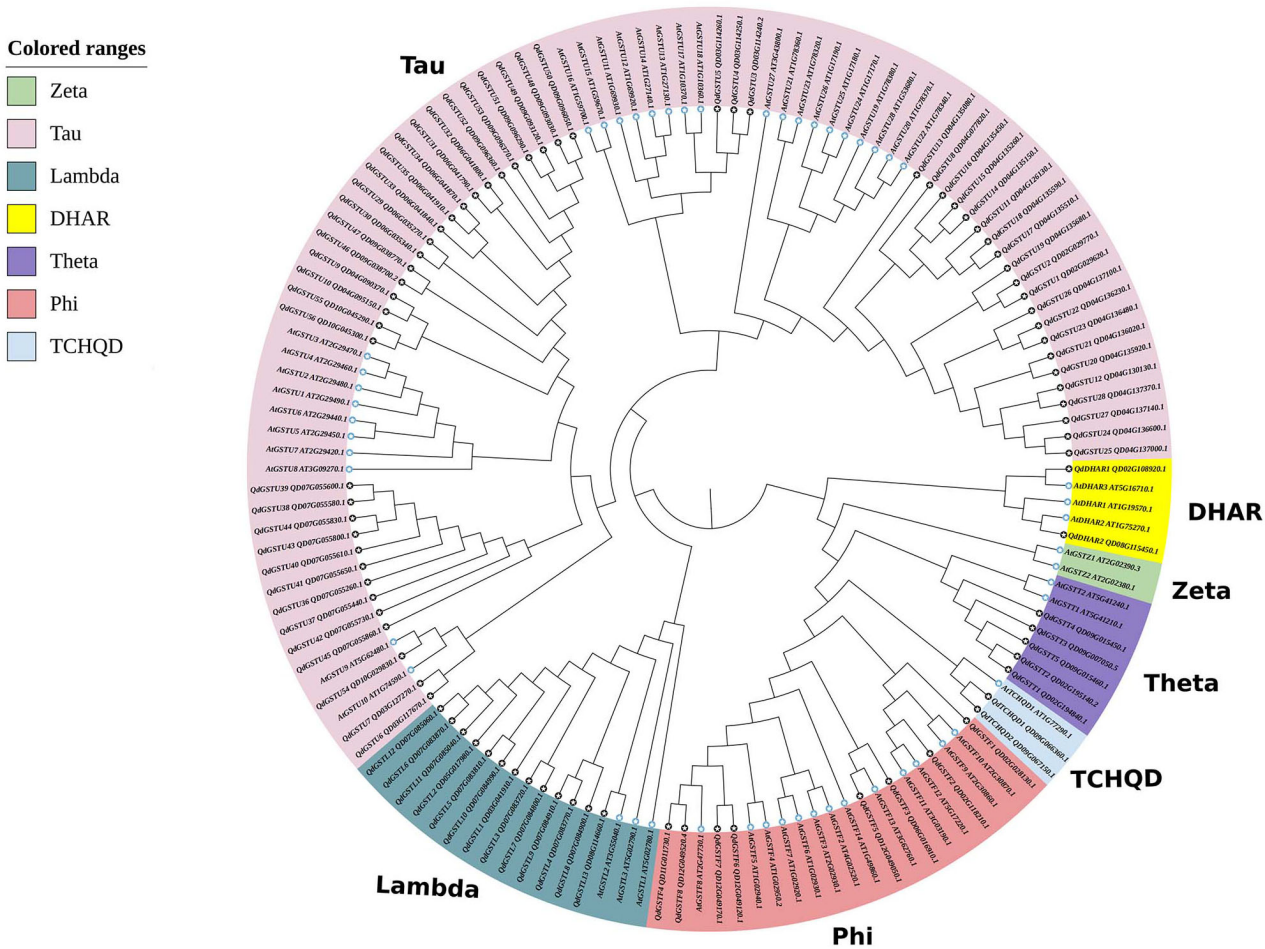
Phylogenetic analysis of GSTs from *Q. dentata* and *A. thaliana*. Different colors represent different classes of GST genes. Species names are abbreviated as follows: At, *A. thaliana*; Qd, *Q. dentata*. Different shapes indicate different species (At, black background star; Qd, blue background star). The tree was constructed using TBtools and the maximum likelihood method with 1,000 bootstrap replications.

Eighty-six *GSTs* in *Q. dentata* were identified and systematically classified on the basis of their chromosomal locations and the homology of their encoded proteins with that of *Arabidopsis* GSTs (**Supplementary Table S1**) (Zhao et al., 2021; Mo et al., 2023). GST proteins from *Q. dentata* (86) and *Arabidopsis thaliana* (56) were used to construct a phylogenetic tree for investigation of the phylogenetic relationships among the QdGSTs. QdGSTs belonging to six classes (tau, lambda, phi, TCHQD, theta, and DHAR) were identified (**Figure 1**), with the majority belonging to the tau class (56 members, 65.1%), followed by the lambda (13 members, 15.1%), phi (eight members, 9.3%), and theta (five members, 5.8%) classes. Only two members each belonged to the TCHQD and DHAR classes, and no QdGST belonged to the zeta class (**Figure 1**).

The coding DNA sequences of *QdGSTs* varied in length from 426 bp (*QdGSTU39*) to 1467 bp (*QdGSTT1*), encoding proteins that ranged from 141 to 488 amino acids. The molecular weights (MWs) of the QdGSTs ranged from 15.9 kDa (QdGSTU39) to 55.1 kDa (QdGSTT1), and their isoelectric point values ranged from 4.48 (QdGSTL1) to 9.38 (QdTCHQD1 and QdTCHQD2). The grand average of hydropathicity values of all QdGSTs, except for QdGSTU3 (0) and QdGSTU54 (0.133), were negative and ranged from −0.02 (QdGSTU24) to −0.57 (QdMTP8.3). Most of the QdMTPs had weak hydrophilicity, and the majority were predicted to have cytoplasmic localization, with limited numbers in the chloroplast and the nucleus (**Supplementary Table S1**).

### 3.2 Chromosomal distribution and duplication of *QdGST* family members

**Figure 2:**
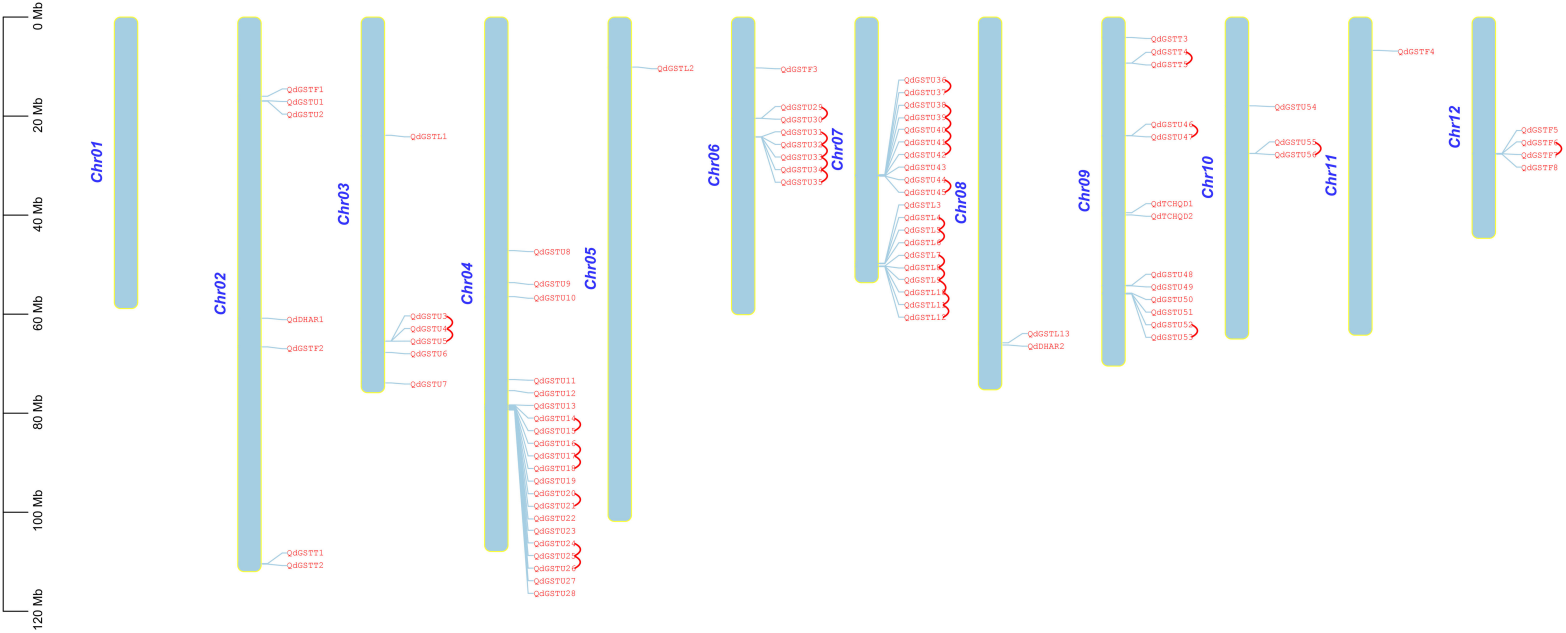
Chromosomal localization of *QdGST* genes in *Q. dentata*. Sky-blue vertical bars represent the *Q. dentata* chromosomes. The chromosome number is shown on the left side of each chromosome. Tandem duplication gene pairs are marked by red curves. The size of the chromosome is indicated by a vertical scale bar.

**Figure 3:**
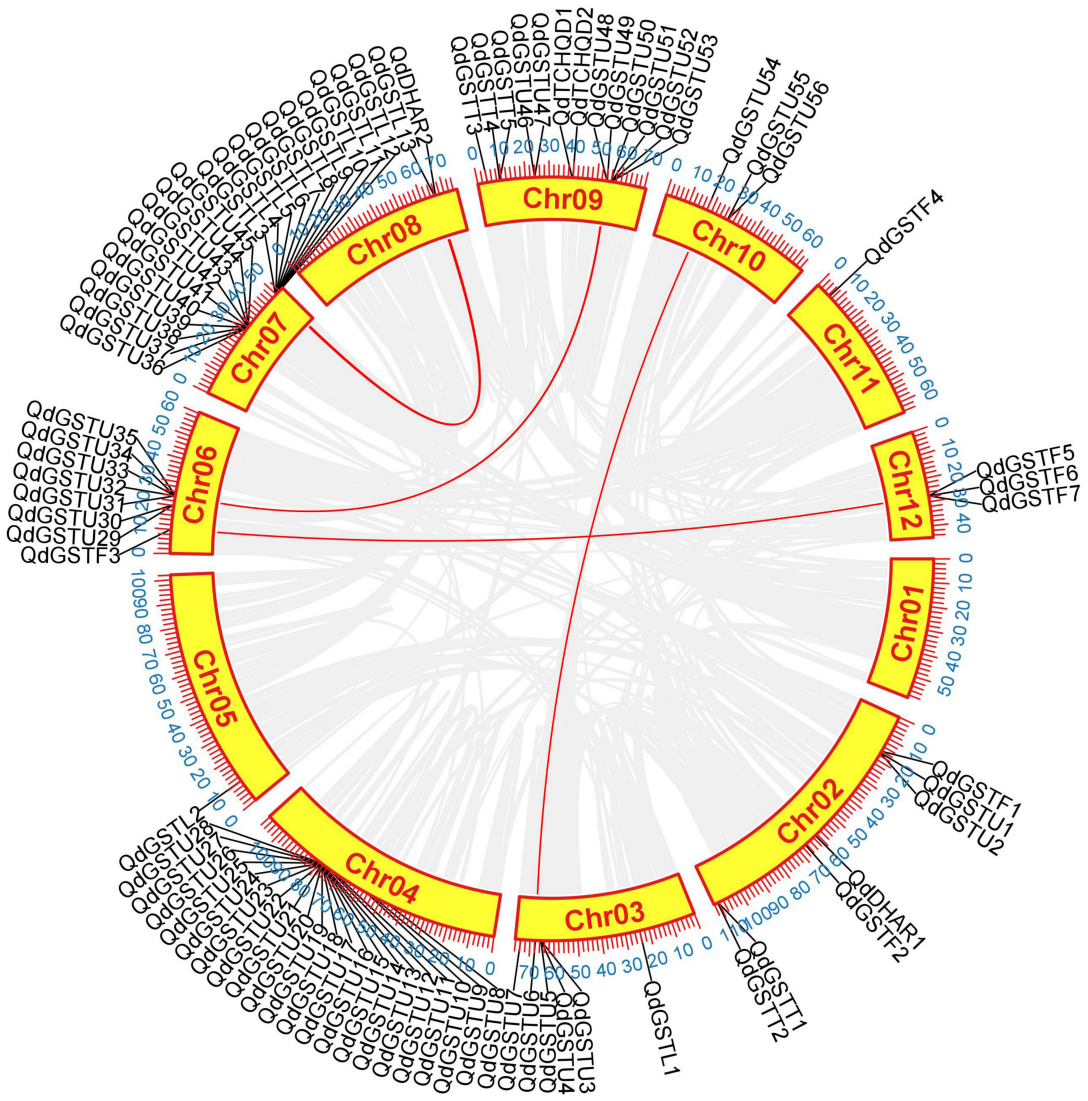
Segmental duplication analysis of *QdGST* genes. Segmental duplication of *QdGST* is shown using a Circos plot. Red curves represent segmental duplications of the GSTs. The gray background shows the genomic positions of all the collinear gene pairs in *Q. dentata*.

The 86 *QdGST* genes were unevenly dispersed across 11 of the 12 chromosomes. Twenty-one (24.4%) and 20 (23.3%) genes, belonging to the tau and lambda members, were located on chromosomes 4 and 7, respectively; chromosome 9 contained 13 *QdGST* genes; and chromosomes 6, 2, 3, 12, 10, and 8 had 8, 7, 6, 4, 3, and 2 *QdGST* genes, respectively. Chromosomes 5 and 11 contained only one *QdGST* gene each (**Figure 2**). No significant association was observed between chromosomal length and number of *QdGST* genes (**Figure 2**). Segmental and tandem duplication have important roles in driving the expansion of gene families (Wang et al., 2019). A total of 31 gene pairs distributed on seven chromosomes were identified as tandem duplication types; 22 of these (71.0%) involved genes from the tau class, and seven (22.6%) involved genes from the lambda class. There was one tandem duplication (0.03%) corresponding to each of the theta and phi classes (**Figure 2**). In addition, seven *QdGST* gene pairs (*QdGSTU6/QdGSTU54*, *QdGSTU31/QdGSTU48*, *QdGSTU34/QdGSTU49*, *QdGSTF3/QdGSTF5*, *QdGSTL3/QdGSTL13*, *QdGSTL7/QdDHAR2*, and *QdGSTL7/QdGSTL13*), located on chromosomes 3, 6, 7, 8, 9, 10, and 12, respectively, were identified as segmental duplications (**Figure 3**). Three of these gene pairs were from the tau class, two from the lambda class, and one from the phi class. Notably, one segmental duplication pair (*QdGSTL7/QdDHAR2*) contained genes from both the lambda and DHAR subfamilies. *QdGSTU31*, *QdGSTU34*, and *QdGSTL7* underwent both segmental and tandem duplication. Overall, these results suggest that the duplication events identified here contributed primarily to the expansion of the tau and lambda classes of *GST* genes in *Q. dentata*.

### 3.3 Analysis of GST gene collinearity

**Figure 4:**
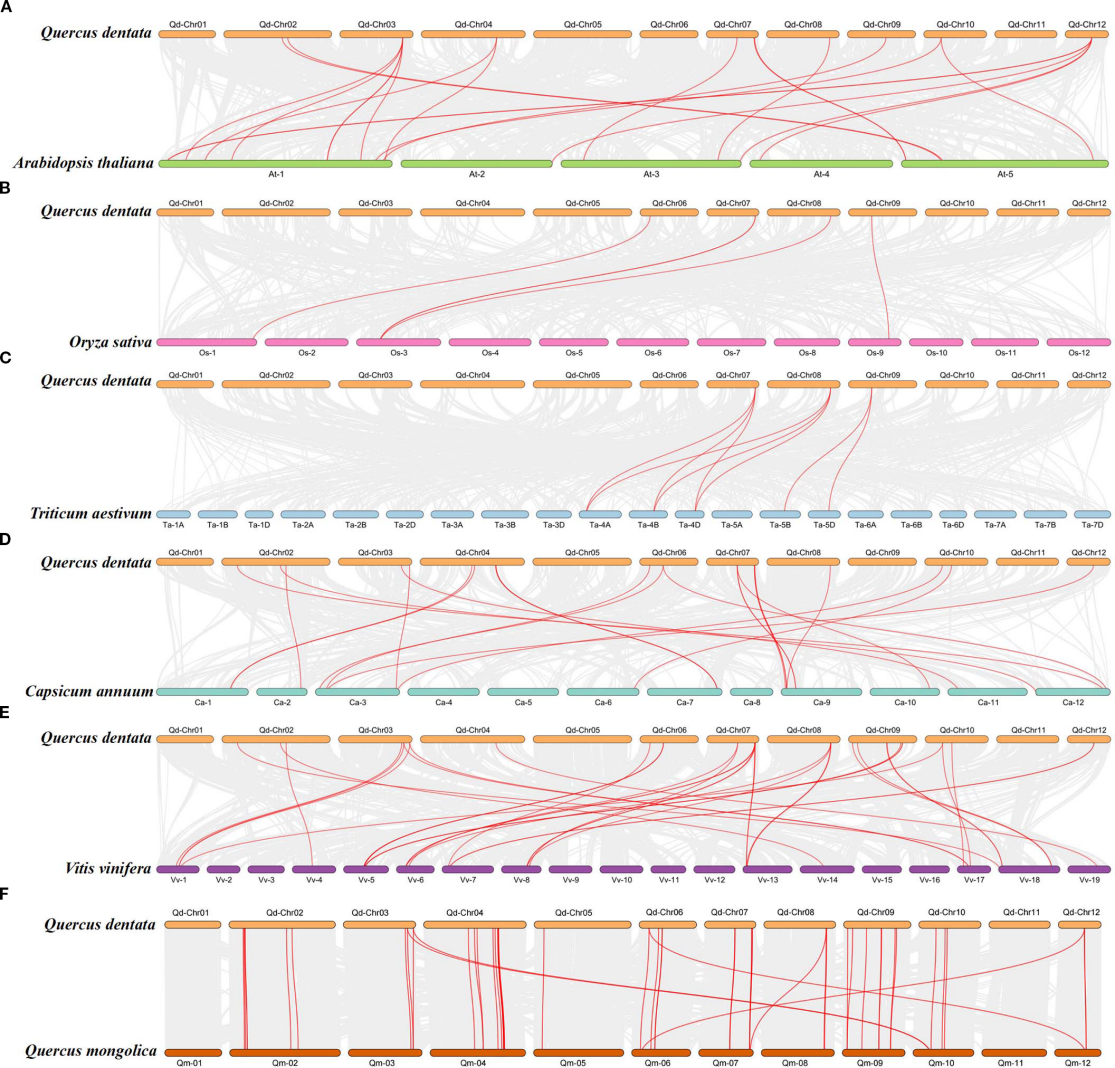
Collinearity analysis of GST genes in *Q. dentata* and six other species: **(A)** *A*) *thaliana*, **(B)** *O. sativa*, **(C)** *T. aestivum*, **(D)** *C annuum*, **(E)** *V. vinifera*, **(F)** *Q. mongolica.* Representative homologous GST gene pairs (red) are overlaid on the collinear gene pairs (gray).

To explore the evolutionary relationships of GST genes in *Q. dentata* and other species, we constructed a synteny map based on six species (*A. thaliana*, *O. sativa*, *C. annuum*, *T. aestivum*, *V. vinifera*, and *Q. mongolica*) and previously published genome-wide GST data (Wang et al., 2019; Islam et al., 2019; Li et al., 2022). We identified 135 orthologous GST gene pairs between *Q. dentata* and the six angiosperm species (**Figure 4**). Of these, 42 collinear blocks of *QdGST* genes were found between *Q. dentata* and *Q. mongolica*, followed by 38 between *Q. dentata* and *V. vinifera*, 23 between *Q. dentata* and *C. annuum*, and 20 between *Q. dentata* and *A. thaliana*. In addition, eight and four orthologous GST gene pairs were detected for *T. aestivum* and *O. sativa*, respectively (**Figure 4**). No *QdGST* gene syntenic regions were found on chromosome 5 or 11 of the *Q. dentata* genome. Three *QdGST* (*QdGSTL13*, *QdGSTL7*, and *QdGSTU46*) gene pairs were detected between *Q. dentata* and two monocot plants (*T. aestivum* and *O. sativa*); this indicates that the majority of *QdGST* genes were formed after the divergence of their common ancestor. Notably, 36 *QdGST* genes lacked detectable orthologs across other species.

### 3.4 Examination of *QdGST* gene structures and conserved protein motifs

**Figure 5:**
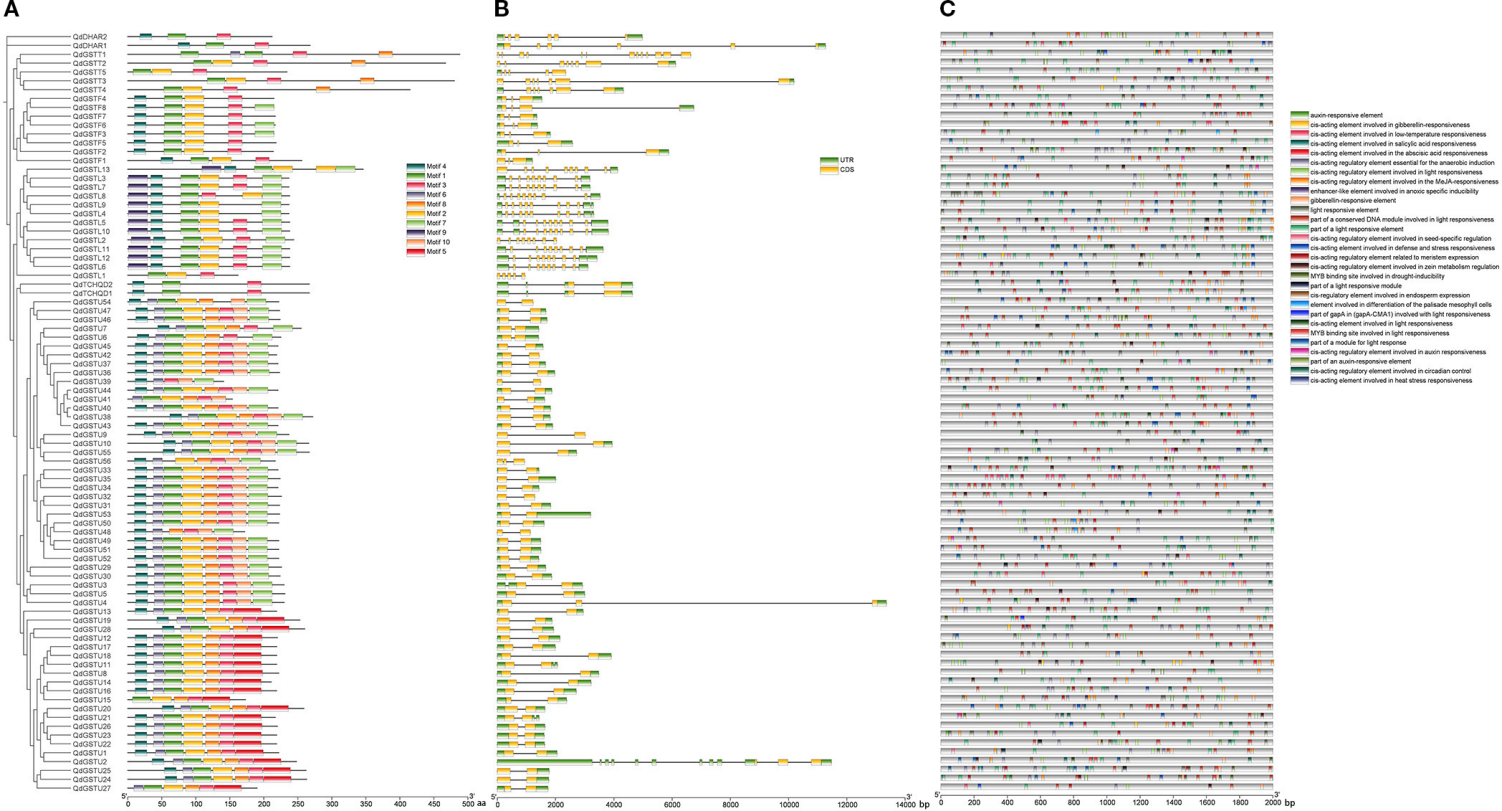
Conserved protein motifs, gene structures, and *cis*-acting elements of *QdGST* genes. **(A)** Composition and distributions of conserved motifs in the *QdGST* genes. Different motifs are shown in boxes of different colors. **(B)** Exon–intron organization of *QdGST* genes. **(C)** Prediction of *cis*-acting elements in the promoters of *QdGST* genes. Different colored boxes indicate different regulatory elements.

We analyzed the composition of QdGST proteins and found ten conserved motifs among the 86 QdGSTs. The presence of these motifs varied among QdGST from different classes, with motif 9 found only in the lambda class, whereas motifs 5 and 10 were specific to the tau class. Motifs 4, 1, and 3 were consistently present in all proteins of the DHAR and TCHQD classes; all members of the theta class had motifs 1 and 3; and all members of the phi class had motifs 4, 1, 2, and 3. All members of the lambda class except for *QdGSTL1* harbored motifs 9, 4, 1, 2, and 7; and most members of the tau class (50/56, 89.3%) contained motifs 4, 6, 1, 2, 8, and 3 (**Figure 5A**). To investigate the evolution of the *QdGST* genes, we analyzed their structures using the *Q. dentata* genome annotation file. We found that the number of introns in *QdGST* genes varied from 0 to 12, with genes within the same class exhibiting similar structural patterns. Most *QdGST* genes (49/56) in the tau subfamily had one intron, and five genes had two introns, and the *QdGSTU2* gene had 11 introns. Most members (11/13) of the lambda class had 9–10 introns. Seven of eight *QdGST* genes in the phi class had two introns; the exception was *QdGSTF8*, which had three. TCHQD- and DHAR-class genes contained three and five introns, respectively, and intron numbers in the theta class ranged from four (*QdGSTT1*) to 12 (*QdGSTT5*) (**Figure 5B**).

### 3.5 Identification of *cis*-regulatory elements of *QdGST* genes

We screened the 2,000-bp promoter regions upstream of the translation initiation sites of the 86 *QdGST* genes using PlantCARE to detect *cis*-acting elements. The results revealed 1,924 *cis*-regulatory elements that could be classified into 29 categories (**Figure 5C**) on the basis of their functions. These functions included response to light, plant hormones, stresses, and plant development. More than 50% of the identified elements were related to light response; these included ACE, G-box, 4cl-CMA2b, MRE, Box 4, ATCT-motif, ATC-motif, chs-CMA2a, AT1-motif, AE-box, and ACA-motif, which were ubiquitously present in the promoter regions of all *QdGSTs* (**Figure 5C**, **Supplementary Table S2**).

A total of 527 *cis*-acting elements were found to be related to signaling by plant hormones, including gibberellin (TATC-box, P-box, GARE-motif), salicylic acid (TCA-element), abscisic acid (ABRE), auxin (TGA-element, AuxRR-core, AuxRE, and TGA-box), and methyl jasmonate (TGACG-motif and CGTCA-motif), as well as zein metabolism regulation (O2-site). A total of 326 *cis*-regulatory elements were implicated in stress responses such as anaerobic induction (ARE, GC-motif), drought response (MBS), low-temperature response (LTR), defense and stress response (TC-rich repeats), and heat response (HSE). Twenty-four and 83 *cis*-acting elements were related to circadian control and plant development (CAT-box, GCN4_motif, RY-element, and HD-Zip 1), respectively (**Figure 5C**, **Supplementary Table S2**).

### 3.6 Expression profiles of *QdGST* genes based on RNA-seq data

**Figure 6:**
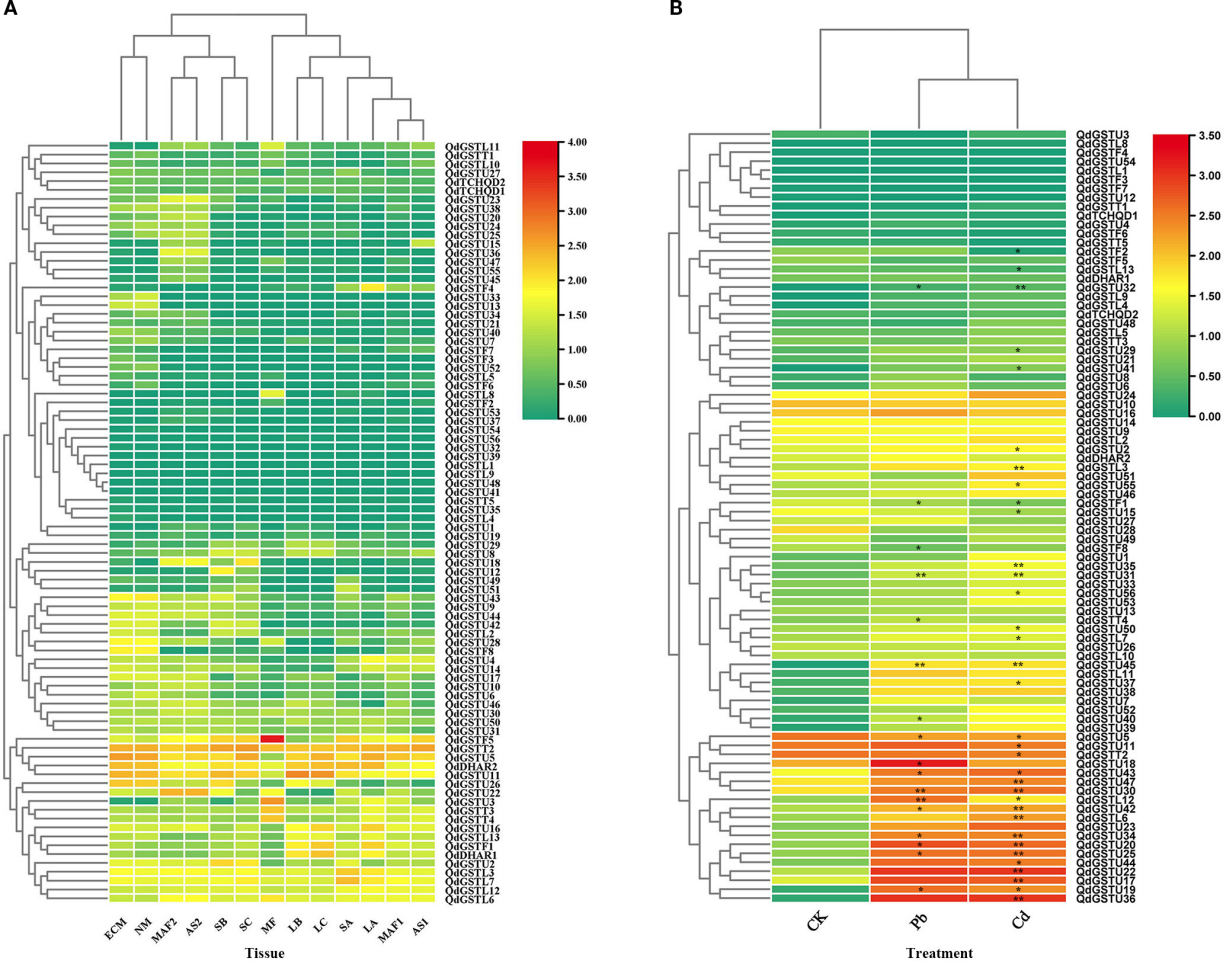
Analysis of expression profiles of *QdGST* genes. **(A)** Heatmaps of the expression profiles of *QdGST* genes in various tissues of *Q. dentata*, including ectomycorrhiza (ECM), root (NM), fruit (MF, MAF1 and MAF2), shell (AS1 and AS2), leaves (LA, LB, and LC), and stems (SA, SB, and SC). **(B)** Expression patterns of *QdGST* genes in roots under Cd and Pb treatments. Black asterisks denote statistically significant differences between heavy metal treatment and control groups (*p < 0.05; **p < 0.01). The color bar represents log_10_(FPKM+1).

The expression patterns of *QdGST* genes were characterized using transcriptome sequencing data from diverse tissues and developmental stages. We detected expression (log_10_(FPKM+1) > 0) of all *QdGST* genes except *QdGSTU41* in at least one tissue at each development stage (**Figure 6A**). Nine *QdGST* genes (*QdDHAR2*, *QdGSTL3*, *QdGSTL7*, *QdGSTL12*, *QdGSTL6*, *QdGSTU5*, *QdGSTU11*, *QdGSTT2*, and *QdGSTT3*) showed high expression in all samples (log_10_(FPKM+1) > 1). The eighty-six *QdGST* genes could be divided into three classes according to their tissue-specific expression profiles: (a) *QdGST* genes with extremely low expression in almost all tissues; (b) *QdGST* genes exhibiting low-to-medium expression across different tissues; and (c) *QdGST* genes with high expression in some tissues. These findings were consistent with previous reports (Islam et al., 2019; Hasan et al., 2021).

To investigate the responses of *QdGST* genes to heavy metal stresses, we analyzed their expression profiles under Pb and Cd treatment using data from transcriptome sequencing. Nine *QdGST* genes of the tau class (*QdGSTU19*, *QdGSTU20*, *QdGSTU25*, *QdGSTU30*, *QdGSTU31*, *QdGSTU32*, *QdGSTU34*, *QdGSTU42*, and *QdGSTU43*) and one member of the lambda class (*QdGSTL12*) were upregulated in response to both Pb and Cd treatment. Two members of the tau class (*QdGSTU18* and *QdGSTU40*), one of the theta class (*QdGSTT4*), and one of the phi class (*QdGSTF8*) were upregulated only in response to Pb treatments, whereas 13 tau-class (*QdGSTU2*, *QdGSTU17*, *QdGSTU22*, *QdGSTU29*, *QdGSTU35*, *QdGSTU36*, *QdGSTU37*, *QdGSTU41*, *QdGSTU44*, *QdGSTU47*, *QdGSTU50*, *QdGSTU55*, and *QdGSTU56*) and four lambda-class (*QdGSTL3*, *QdGSTL6*, *QdGSTL7*, and *QdGSTL11*) genes were upregulated only by Cd treatment. *QdGSTU19*, *QdGSTU20*, *QdGSTU36*, *QdGSTU39*, *QdGSTU44*, *QdGSTU45*, and *QdGSTL11* showed particularly marked upregulation (>100-fold increase in expression) under Pb and/or Cd treatment (**Figure 6B**).

### 3.7 Expression of *QdGST* genes under Pb and Cd stresses according to qRT-PCR

**Figure 7:**
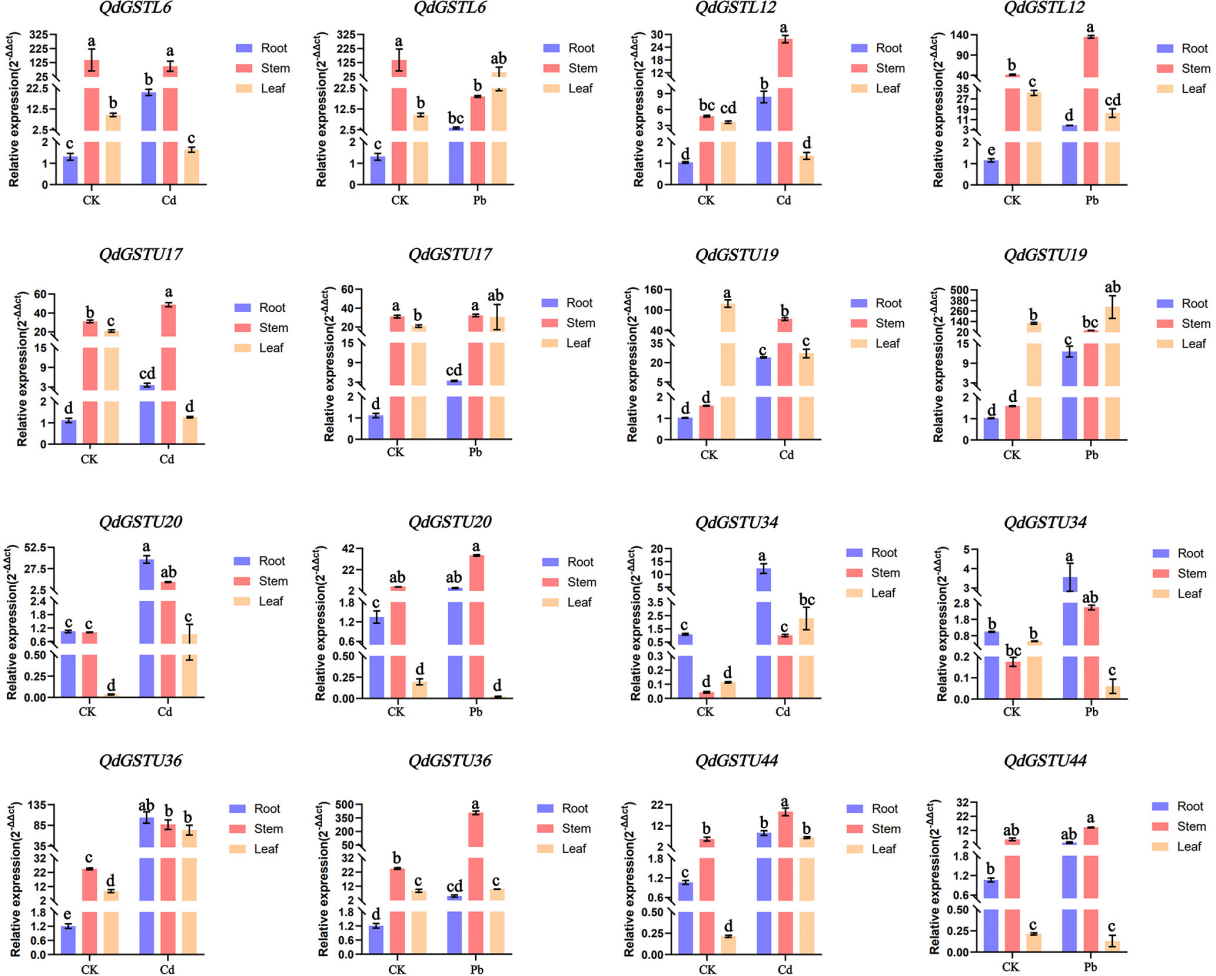
Relative expression patterns of eight *QdGST* genes in *Q. dentata* seedlings under Pb and Cd stresses. Expression was analyzed by qRT-PCR with three biological replicates; the results are presented as the mean ± standard deviation. Untreated controls are denoted CK. Lowercase letters indicate significant differences between control and treatment groups (*p* < 0.05).

The results of the RNA-seq analysis for eight *QdGST* genes with high expression levels under Pb and Cd treatments were validated using qRT-PCR. Under normal growth conditions, the expression patterns of these genes showed tissue specificity, with *QdGSTL6*, *QdGSTL12*, *QdGSTU17*, and *QdGSTU36* having higher levels in stems and leaves than in roots (**Figure 7**), whereas *QdGSTU20* and *QdGSTU44* had higher expression levels in roots and stems compared to leaves, and *QdGSTU19* and *QdGSTU34* were highly expressed in stems and roots, respectively. All the tested *QdGST* genes were upregulated under both Pb and Cd treatments in stems, and all except *QdGSTL6* were upregulated under Cd treatment in roots. Expression of *QdGSTL12*, *QdGSTU19*, *QdGSTU20*, *QdGSTU34*, *QdGSTU36* and *QdGSTU44* was induced by Pb treatment. In leaves, the expression of *QdGSTU20, QdGSTU34*, *QdGSTU36*, and *QdGSTU44* was induced by Cd treatment, whereas that of *QdGSTL6* was upregulated by Pb treatment. However, four *QdGST* genes (*QdGSTL6*, *QdGSTL12*, *QdGSTU17*, and *QdGSTU19*) were inhibited by Cd and three (*QdGSTL12*, *QdGSTU20*, and *QdGSTU34*) by Pb in leaves.

### 3.8 Growth of recombinant yeast under heavy metal stresses

**Figure 8:**
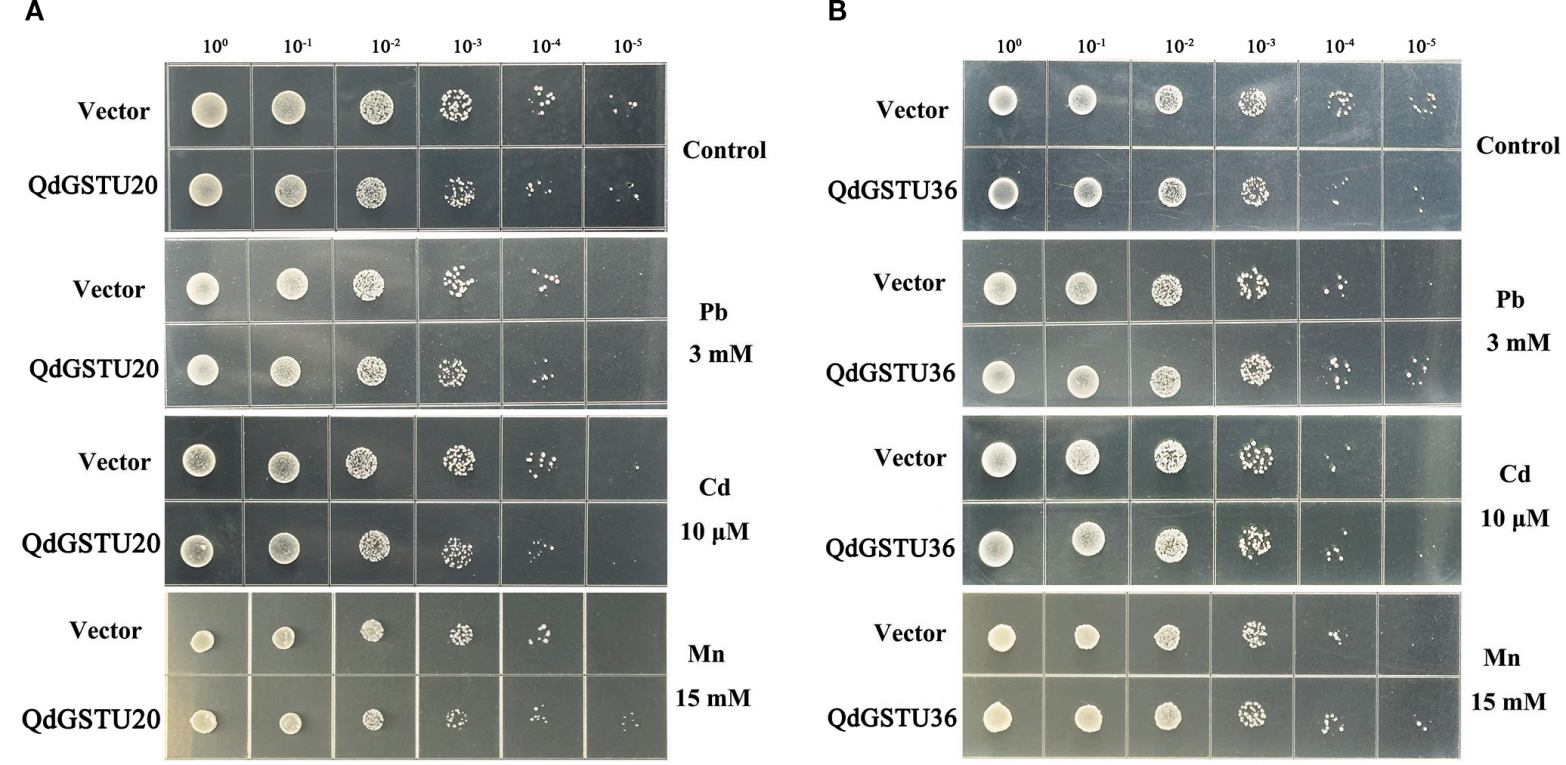
Tolerance of yeast expressing heterologous *QdGST* to heavy metal stresses. *S. cerevisiae* strain INVSc1 was genetically modified to harbor either the empty pYES2 plasmid or a recombinant vector containing the *QdGSTU20* **(A)** or *QdGSTU36* **(B)** gene. Two-microliter serial dilutions of yeast cultures were inoculated onto SD Ura/Gal medium treated with heavy metals.

To further characterize the involvement of *QdGST* genes in heavy metal resistance, *QdGSTU20* and *QdGSTU36*, which were highly expressed under both Pb and Cd treatments, were introduced into wild-type yeast for metal sensitivity analysis. Under normal conditions, yeast strains with empty vectors and those expressing *QdGSTU20* or *QdGSTU36* showed no significant difference in growth. However, when expressed in yeast, *QdGSTU36* conferred Pb and Cd tolerance. Expression of *QdGSTU20* did not alter the sensitivity of yeast to Pb or Cd; however, it did confer Mn tolerance (**Figure 8**).

## 4 Discussion

The GST superfamily represents an evolutionarily conserved group of proteins found across diverse living organisms. Plant GSTs have important roles in regulation of growth and development and improve plant resistance to various stresses (Pinkus et al., 1996; Gullner et al., 2018; Kayum et al., 2018; Shokat et al., 2020). Many *GST* genes have been detected in various plants, with their numbers varying greatly among species. Until now, the GST family in *Quercus* species had not been analyzed. In the present study, we identified 86 GST genes in *Q. dentata*, a number larger than that in rice (Jain et al., 2010), *Arabidopsis* (Sappl et al., 2009), barley (Rezaei et al., 2013), maize (McGonigle et al., 2000), *Capsella rubella* (He et al., 2016), radish (Gao et al., 2020), or pepper (Islam et al., 2019), but smaller than that in wheat (Wang et al., 2019), soybean (Hasan et al., 2020), potato (Islam et al., 2018), litchi (Hu et al., 2016), or tomato (Lan et al., 2009). These *QdGST* genes were found to be distributed among six of the GST classes, with the greatest abundance in the tau class (56 members), followed by the lambda (13) and phi (8) classes. These results are in contrast to those of previous research in other species, which found that the phi- and tau-class GSTs were most abundant, with numbers of lambda-class members ranging from only two to five in many plant species (He et al., 2016; Islam et al., 2019; Wang et al., 2019; Liu et al., 2023). Members of the zeta class have been identified in various monocotyledonous and dicotyledonous plants (Wang et al., 2019). However, we found no QdGST belonging to this class, similar to findings in apple (Fang et al., 2020). Therefore, we speculate that the zeta class of GSTs may have been lost during the evolution of *Q. dentata*.

Gene family expansion depends on various duplication mechanisms, including tandem, segmental, and whole-genome duplications, as well as transpositions (Cannon et al., 2004; Flagel and Jonathan, 2009). Previous studies have shown that tandem and segmental duplications, which are distinctive features of plant genomes, resulted in most of the members of the tau and phi classes of GST (Islam et al., 2019; Hao et al., 2021; Wang et al., 2023). In the present study, 48 *QdGST* genes were found to be associated with tandem duplication, whereas 12 were associated with segmental duplication. These results show that tandem duplication had a significant role in the evolution of *QdGST* genes, consistent with findings in other plant species (Jain et al., 2010; He et al., 2016; Fang et al., 2020). Duplication events can drive the expansion of gene families and generate novel gene functions, which can enable plants to quickly adapt to adverse environmental conditions (Freeling, 2009). In *Q. dentata*, most tandem duplication events occurred between gene pairs in the tau class, resulting in tau QdGSTs being the most common. The tau GSTs also seemed to have essential roles in the tolerance of *Q. dentata* to different stresses. The lambda class was the second largest, owing to both tandem and segmental duplication events. This phenomenon has not been observed in previous studies. Therefore, further research is needed to understand the roles of the expanded lambda class of GSTs in *Q. dentata*.

GST genes of the tau class in plants have been reported to have important roles in adaptation to diverse environmental stresses. For instance, *DsGSTU1* in *Digitaria sanguinalis* is associated with resistance to haloxyfop-*P*-methyl, an acetyl-CoA-carboxylase-inhibiting herbicide (Liu et al., 2023), and ectopic overexpression of a *BcGSTU* gene from *Brassica campetris* subsp. Chinensis in *Arabidopsis* results in better performance of plants under abiotic (NaCl and PEG) and biotic (*Alternaria brassicae* infection) stresses (Kao et al., 2016). Moreover, *OsGSTU17* in *O. sativa* and *MruGSTU39* in *Medicago ruthenica* improve drought stress tolerance (Wang et al., 2022; Li et al., 2023), overexpression of *SbGST* from *Salicornia brachiata* in transgenic tobacco has been found to enhance seed germination and growth under salt stress (Jha et al., 2011), *GmGSTU23* increases salt tolerance in soybean (Li et al., 2023), *PeGSTU58* from *Populus euphratica* enhances salt and drought stress tolerance (Meng et al., 2023), a rice *OsGSTU4* improves tolerance to salinity and oxidative stresses in *Arabidopsis* (Sharma et al., 2014), and *JrGSTTau1* improves plant tolerance to chilling (Yang et al., 2016). Tau-class GST genes are predominant in woody plants, including *V. vinifera* (88 tau GSTs of a total of 132), *Populus trichocarpa* (66/79), *Coffee canephora* (34/54), *Citrus sinensis* (12/25), *Prunus avium* (52/67), *Amborella trichopoda* (36/52), and *Picea abies* (73/104) (Monticolo et al., 2017; Sabir et al., 2022). Woody plants are constantly exposed to various environmental stresses such as drought, extreme temperatures, pathogens, and herbivores. Thus, the expansion of the tau class of GST genes in these plants may represent a key evolutionary innovation underpinning their adaptation to their environments. As in other woody species, the tau class (56 of 86) represents the majority of GSTs in *Q. dentata*. The heavy-metal-inducible expression of *QdGSTU* genes demonstrated here and its functional validation in yeast indicate a critical role of the tau class in adaptation to environmental stress.

With increasing industrialization, contamination of soil by heavy metals is a growing environmental concern (Li et al., 2022). Research has demonstrated that *Quercus* species exhibit significant tolerance to multiple heavy metals (e.g., Sb, Cd, Cu, Pb, Zn, Co, and Ni) and serve as effective agents in phytoremediation of contaminated soils (Gogorcena et al., 2011; Shi et al., 2017, Shi et al., 2019). Although various plant GSTs have been reported to respond to exposure to different heavy metals (Sappl et al., 2009; Lin et al., 2013; Kumar et al., 2013), the role of the *Quercus* GST family remained unclear. Here, we found that 31 GST genes in *Q. dentata* were upregulated under Cd or Pb treatment, most of which were members of the tau class. In particular, expression levels of tau-class *QdGSTU19*, *QdGSTU20*, *QdGSTU36*, and *QdGSTU44* were strongly upregulated under Pb and/or Cd treatment according to our RNA-seq analysis; these results were validated by qRT-PCR. Our findings are consistent with those previously reported in other plant species (Lin et al., 2013; Gao et al., 2020; Jing et al., 2020), confirming that GST genes of the tau class have important roles in plant tolerance to heavy metal stress. Heterologous expression of *QdGSTU36* in yeast conferred the ability to tolerate Cd and Pb stresses, consistent with the expression profiles of this gene obtained in the qRT-PCR analysis. However, heterologous expression of *QdGSTU20* provided tolerance only to Mn. We hypothesize that expansion of the tau class has enabled *Q. dentata* to develop more precise adaptation mechanisms under complex environmental conditions. Our results provide candidate genes for future development of heavy-metal-tolerant plants, with *QdGSTU36* and *QdGSTU20* showing particular importance in this regard. However, the lack of in-plant validation (e.g., overexpression/knock-out studies of *QdGST* genes in *Arabidopsis* or *Q. dentata*) limits the biological relevance of these findings to actual plant stress responses. Therefore, future work should include generation of stable transgenic *Arabidopsis* or *Q. dentata* plants via over-expression, CRISPR–Cas, or RNA interference; these could be used to (a) evaluate growth and physiological parameters under heavy metal stresses; and (b) quantify metal accumulation and localization. Integrating the results of these plant assays with our yeast data will provide a more complete understanding of how tau-class GSTs mediate heavy-metal detoxification in *Quercus* plants.

Previous research has shown that GST gene expression is regulated by multiple signaling molecules, including reactive oxygen intermediates (hydrogen peroxide) and plant growth regulators such as auxins, salicylic acid, ethylene, jasmonic acid, and nitric oxide (Moons, 2005). Several upstream regulators of GST genes have been identified through yeast one-hybrid and dual luciferase reporter assays. For instance, *JrDREB2A*, *JrMYC2*, *JrMYB44*, *JrDof1*, and *JrWRKY7* were shown to directly activate *JrGSTTau1* expression to regulate osmotic stress response in *Juglans regia* (Yang et al., 2019); *LhGST*, which belongs to the phi class, was found to be crucial for anthocyanin transport and accumulation in lily tepals, and its promoter could be activated by *LhMYB12-lat* (Cao et al., 2021); similarly, *MrGST1*, another phi-class GST, was activated by *MrMYB1.1* and regulated anthocyanin accumulation in Chinese bayberry (*Morella rubra*) fruit (Xue et al., 2022). In the present study, our analysis of the promoter sequences of *QdGSTU36* and *QdGSTU20* showed the presence of salicylic acid-, abscisic acid-, and methyl jasmonate-responsive elements, as well as an MYB recognition site. These results indicate potential roles of plant hormone signaling in the heavy metal tolerance of *Q. dentata*, including interactions with GST genes. However, further experiments are need to clarify the pathways contributing to heavy metal stress tolerance in *Q. dentata*.

## 5 Conclusions

A total of 86 GST genes were identified in the *Q. dentata* genome and found to comprise members of six classes, with an uneven distribution across 11 chromosomes. Promoter analysis revealed the presence of 29 categories of *cis*-acting elements, most of which were involved in defense and stress responses. RNA-seq and qRT-PCR analyses demonstrated that the *QdGST* genes of the tau class are crucial for heavy metal tolerance; when expressed in yeast, *QdGSTU36* conferred Cd and Pb tolerance, whereas *QdGSTU20* conferred Mn tolerance. These findings lay a foundation for further functional verification of *QdGST* genes. Moreover, they provide a basis for genetic engineering to develop heavy-metal-tolerant crops via overexpression of *QdGST* genes or CRISPR-based genome editing.

## Data Availability

The datasets presented in this study can be found in online repositories. The names of the repository/repositories and accession number(s) can be found in the article/**Supplementary Material**.
